# Endovascular Approach in Treating Vertebrobasilar Insufficiency: A Systematic Review

**DOI:** 10.7759/cureus.56479

**Published:** 2024-03-19

**Authors:** Fakhri Awawdeh, Varun Soti

**Affiliations:** 1 Neurological Surgery, Lake Erie College of Osteopathic Medicine, Elmira, USA; 2 Pharmacology and Therapeutics, Lake Erie College of Osteopathic Medicine, Elmira, USA

**Keywords:** basilar artery occlusion, vertebral artery stenosis, stent, endovascular surgical repair, vertebrobasilar insufficiency (vbi)

## Abstract

Vertebrobasilar insufficiency (VBI) is a significant medical condition that results from a lack of adequate blood flow to the posterior circulation of the brain. The first-line treatment involves the use of antiplatelet therapy, but in cases where patients are not responsive to drug therapy, surgical management is the next viable option. In the past, open endarterectomy was the preferred surgical approach for treating critical VBI patients. However, due to its high mortality rates and severe peri-procedural complications, its usage has decreased. Instead, the endovascular approach has emerged as an alternative surgical option for resolving VBI. This review explores the current literature to assess the effectiveness of endovascular interventions in treating VBI patients. It also highlights potential complications and adverse effects associated with these treatments while identifying gaps in the current research that warrant further investigation. The review adhered to the Preferred Reporting Items for Systematic Reviews and Meta-Analyses (PRISMA) guidelines to extensively search relevant literature on endovascular approaches for treating VBI patients on PubMed, BioMed Central, and ClinicalTrials.gov. The findings suggest that endovascular treatments have demonstrated significant technical success in treating VBI, with low mortality rates and minor adverse effects, such as intracranial hemorrhage and restenosis. The overall incidence of these complications is relatively low. Combining medical therapies with endovascular interventions has improved outcomes and reduced restenosis rates. However, there are methodological limitations and inconsistencies in the current literature that necessitate further investigation. Future research should focus on larger, randomized clinical trials and direct comparisons with other treatment options to obtain more conclusive evidence.

## Introduction and background

Vertebrobasilar insufficiency (VBI) results from inadequate blood flow to the brain's posterior portion. Two vertebral arteries (VA) supplying this portion merge and form the basilar artery. The posterior circulation supplies blood to many significant areas including the brainstem, thalamus, hippocampus, cerebellum, and occipital and medial temporal lobes. VBI often occurs due to atherosclerosis secondary to cholesterol build-up, pulmonary embolism, and penetrating artery disease among many other conditions [1].

Patients with VBI are at an extremely high risk for stroke or transient ischemic attack. Furthermore, patients older than 50 with comorbidities such as coronary artery disease, diabetes, obesity, smoking, hyperlipidemia, and family history are at a higher risk [1]. As many as 25% of patients over the age of 70 may have VBI. Moreover, patients with VBI tend to have a multitude of symptoms including but not limited to ataxia, dizziness, vertigo, double vision, dysphagia, and slurred speech [2]. 

Digital subtraction cerebral angiography is the gold standard to diagnose VBI. The other modalities include computed tomography angiography (CTA), magnetic resonance angiography (MRA), and single photon emission computed tomography. Vertebrobasilar artery (VBA) stroke may present with moderate to significant episodic vertigo induced by flexion or extension of the cervical spine. Therefore, obtaining an X-ray, CTA, or MRA is vital to rule out cervical pathology [3]. Besides, transcranial Doppler ultrasound, performed at the bedside, can assess the hemodynamics of the cerebral vessels if warranted in patients with VBI [2]. 

VBI treatment is complex, and over the years, surgeons have been developing techniques and surgical approaches that are more minimally invasive, produce better outcomes with shorter operating room times, and are more cost-effective. Traditionally, surgeons would likely opt for endarterectomy in such patients, and research shows that the five-year survival rate is 67.6% after carotid endarterectomy [4]. In contrast, treatment through endovascular repair has a 93% success rate. Still, in the last decade, endovascular techniques, such as endovascular repair, have advanced considerably in treating VIB patients [5]. 

Endovascular repair with stenting offers a promising alternative. The procedure involves tailoring the puncture site to the patient's anatomy. Typically, surgeons opt for the femoral artery as the puncture site. However, the radial and brachial arteries are also viable options. A 6-French (Fr) or 7-Fr Judkins catheter (for VA) or an internal mammary artery catheter (for subclavian artery) serves as the guiding catheter. Using a buddy wire in the distal subclavian artery provides additional stability. A 0.014-inch steerable guidewire passes through the lesion [6]. 

The endovascular repair offers several advantages, including a shorter recovery time, less discomfort, local or regional anesthesia instead of general anesthesia, smaller incisions, reduced strain on the heart, and reduced risks for patients with other medical conditions. Moreover, it is a highly effective and feasible approach for elderly patients with multiple comorbidities [6]. 

VBI remains a rare complication often underreported or misdiagnosed. Moreover, endarterectomy, the most widely used surgical option, has yet to galvanize most surgeons in its support due to insufficient evidence of its effectiveness in treating symptomatic VIB patients despite being in clinical practice for so long. Therefore, this review explores the current literature to assess the effectiveness of endovascular interventions in treating VBI patients. It also highlights potential complications and adverse effects associated with these treatments while identifying gaps in the current research that warrant further investigation. This article sheds light on the robustness of endovascular repair, an alternate surgical modality to treat VBI patients to promote awareness and help healthcare practitioners make better surgical choices for the best patient outcomes.

## Review

Literature search, study selection, and data extraction

**Figure 1:**
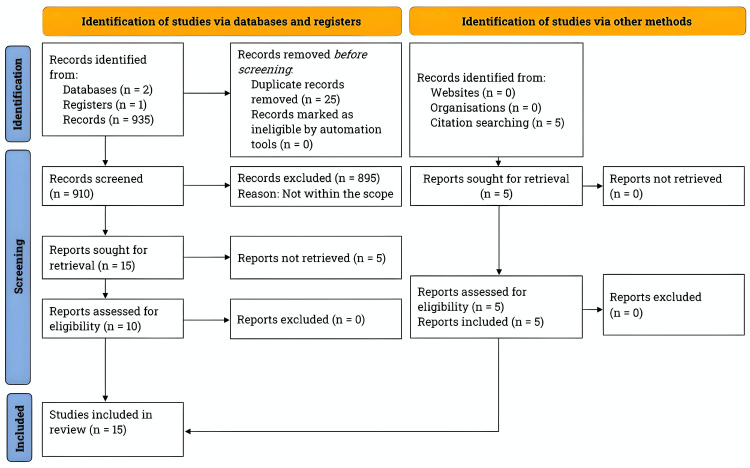
Literature search and study selection process. We followed the PRISMA guidelines for the literature search. We thoroughly searched for studies on the effectiveness of the endovascular approach in treating vertebrobasilar insufficiency using PubMed, BioMed Central, and ClinicalTrials.gov. The search was conducted between January and December 2023 and focused on studies published in the last 20 years. n: number; PRISMA: Preferred Reporting Items for Systematic Reviews and Meta-Analyses

We conducted a comprehensive literature search from January to December 2023, following Preferred Reporting Items for Systematic Reviews and Meta-Analyses (PRISMA) guidelines [7]. Please refer to Figure 1 for the PRISMA flowchart outlining this review's literature search and study selection process.

Our investigation focused on PubMed, BioMed Central, and ClinicalTrials.gov to retrieve primary trials assessing the effectiveness of endovascular repair and open endarterectomy in treating patients with VBI. Specifically, we extracted the studies conducted in the last 20 years. Please refer to Table 1 for the criteria for study selection.

**Table 1:** Study selection criteria. We included studies published in English that focused on patients with vertebrobasilar insufficiency managed through an endovascular approach and met the inclusion criteria mentioned above.

Inclusion criteria	Exclusion criteria
Randomized controlled trials	Pre-clinical studies
Non-randomized controlled trials	Systematic reviews
Prospective studies	Narrative reviews
Pilot studies	Commentaries
Observational studies	
Retrospective studies	
Meta-analysis	
Case series	
Case report	

After identifying the relevant studies, we assigned each study a level of clinical evidence based on existing literature [8]. To extract data, we used search terms such as "Vertebrobasilar Insufficiency," "Vertebrobasilar Insufficiency AND Diagnosis," and "Vertebrobasilar Insufficiency AND Endovascular Repair." After a meticulous review, we carefully selected the most relevant studies within the scope of this paper.

Surgical technique for endovascular repair

The endovascular procedure requires the use of a guidewire. A guide or sheath approach is most suitable for treating the VA segments V1-V3 stenosis. An 8-Fr system is typical, although a 6-Fr or 7-Fr system may be ideal for a coronary balloon-expandable stent. A standard hydrophilic guidewire and a 6-Fr guide catheterize the target subclavian artery. The guide catheter should advance to the proximal VA origin, providing sufficient stability. For a tortuous subclavian artery, keeping a 0.014-inch buddy wire or a large-caliber coronary guiding catheter may be necessary for support [9].

Next, obtain the biplane road map images and cross the stenosis using a curved-tip 0.014-inch or 0.018-inch guidewire. The curved tip helps navigate the stenosis and prevents subintimal dissection in the stenotic or distal segments within the VA. Position the wire tip in the distal cervical VA, ensuring it is within the field of view on the fluoroscope. Surgeons can alternatively use an embolus protection device [9].

Subsequently, determine the degree of stenosis concerning the diameter of the unaffected vessel segment immediately distal to the stenosis. Once that is determined, proceed to place the stent. The length of the stent should be long enough to extend approximately 1 millimeter (mm) to 2 mm (about 0.08 inches) into the lumen of the ipsilateral subclavian artery and at least 3 mm (about 0.12 inches) into the normal distal VA, ensuring the coverage of the entire lesion. After positioning the stent, perform an angiogram in the working projection (used for stent deployment) to document the technical result of the procedure. Finally, compare this angiogram with the initial pre-procedure angiogram [9].

In some cases, angioplasty with a small balloon may be necessary for very tight stenosis to allow better positioning of the definitive balloon stent system. However, using coronary balloon-expandable stents for treating stenosis in the VA origin is routine due to their placement accuracy. These stents have adequate radial force, a low crossing profile, and limited foreshortening. Surgeons often choose coronary balloon-expandable stents for stenosis in the V2 segment because of its fixed bony location [9].

Additionally, there are self-expanding stents available for treatment. For instance, due to vessel tortuosity, a nitinol self-expanding stent is suitable for stenosis involving the V3 segment. Nevertheless, self-expanding stents have noteworthy drawbacks, such as limitations in diameter size. Moreover, the occasional misplacement of self-expanding stents might necessitate the placement of an extra stent [10]. Hence, coronary stents are the preferred option for severely tortuous vessels where support might be a concern. The choice between monorail or over-the-wire systems depends on the surgeon's experience and comfort level.

In recent years, surgical practice has witnessed an increase in the utilization of drug-eluting stents (DES) coated with sirolimus or paclitaxel in diabetic patients. DES reduces restenosis by inhibiting smooth muscle and endothelial proliferation. Although extensively supported in coronary literature, DES has recently been associated with cases of clot formation, leading to stent site thrombosis [11]. However, it is essential to note that limited data on using DES in the VA is available.

Clinical evidence on the effectiveness of endovascular repair in treating VBI patients

Antoniou et al., in a comprehensive analysis, determined the outcomes of endovascular treatment in patients with proximal VA stenosis. They reported on percutaneous transluminal angioplasty (PTA) or stenting of 1117 VA performed in 1099 patients. The majority of patients presented with contralateral VA disease, although 484 patients had bilateral VA disease. All patients experienced symptoms related to the posterior circulation along with persisting VBI symptoms that did not respond to conservative medical management. Most patients received a combination of aspirin and clopidogrel as dual antiplatelet therapy. The treatment was initiated several days before the endovascular procedure and maintained for one to 12 months following the surgery. Therapy with a single antiplatelet medication was continued during the study follow-up [12].

The researchers described the technical success rate as achieving less than 20% residual stenosis of the treated VA segment at the end of the procedure. Most surgeons decided to intervene when stenosis levels exceeded 70%. The study results showed that the endovascular surgical procedure had a remarkable success rate of 97%. There was a minimal occurrence of adverse effects, with only 17 patients (1.5%) experiencing a transient ischemic attack during the procedure. Stroke and death were reported, but the combined rate was low at 1.1%. Among the 967 patients who had a follow-up between six and 54 months after the procedure, 65 (7%) reported recurrent VBI symptoms. Restenosis was observed in 183 out of 789 patients (23%) who underwent follow-up imaging studies. However, only 86 out of 967 patients (9%) required reintervention during the follow-up period [12].

In a multicenter, randomized, open-label clinical trial, Liu et al. investigated the effectiveness and safety of an endovascular surgical approach for treating acute strokes caused by VBA occlusion. The researchers divided the patients into two groups: the intervention group, where patients underwent an endovascular procedure along with standard treatment, and the control group, where patients received standard treatment alone. Of the 288 patients assessed for eligibility, 133 were randomly assigned to the two groups, with 66 in the intervention group and 65 in the control group [13].

The primary goal of the study was to determine the number of patients who achieved a modified Rankin Scale (mRS) score of 0-3 at 90 days follow-up. An mRS score of 0 means the absence of symptoms, while a score of 1 indicates the ability to perform all usual activities. A score of 2 signifies the ability to manage personal affairs without assistance, although not all previous activities can be done. A score of 3 reflects the need for help but the ability to walk unassisted. The secondary clinical endpoint was functional independence, defined as an mRS score of 0-2 at 90 days. Additionally, the researchers monitored 90-day mortality as the primary safety endpoint, along with symptomatic intracranial hemorrhage as a secondary safety measure [13].

The study findings demonstrated that patients in the intervention group had comparable mRS scores between 0 and 3 to those in the control group. In the intervention group, 28 out of 66 patients (42%) achieved this outcome, compared to 21 out of 65 patients (32%) in the control group (p=0.23). Regarding the secondary clinical endpoint, 22 out of 66 patients (33%) in the intervention group achieved functional independence with mRS scores between 0 and 2 at 90 days. This result was statistically comparable to the control group (p=0.48), where 18 out of 65 patients (28%) gained functional independence within the same timeframe. Although there was no statistical difference in the 90-day mortality rate between the groups (p=0.54), the intervention group had a higher numerical incidence of symptomatic intracranial hemorrhage. Five out of 66 patients (8%) in the intervention group experienced this complication, compared to none in the control group. However, this difference was not statistically significant (p=0.06). The results indicated the endovascular approach was effective and safe in treating patients with VBA occlusion and was on par with the standard therapy [13].

PTA of the intracranial VBA is known to carry serious risks, such as arterial dissection and acute occlusion, as well as restenosis after surgery. Studies suggest that around 20% of cases experience peri-procedural complications, while restenosis occurs in approximately 27% of cases. However, Kiyosue et al. offered a promising solution in the endovascular approach. They demonstrated the effectiveness of endovascular stenting in preventing these complications through a detailed case report of two patients [14].

In both cases, the researchers successfully treated restenosis of the intracranial VBA after PTA using flexible coronary stents via an endovascular approach. Notably, no complications arose during or after the procedure, and patients exhibited no signs of restenosis or recurrent symptoms within the four- to six-month follow-up period. The researchers emphasized the substantial benefits of endovascular stenting in reducing the risk of arterial dissection and restenosis during the treatment of intracranial VBA. This breakthrough holds the potential for improving patient outcomes and providing a safer surgical method [14].

A study conducted by Li et al. further supported the efficacy of endovascular techniques in treating symptomatic VA-origin stenosis. Through a retrospective analysis, the researchers examined clinical and angiographic records of patients diagnosed with this condition. All of the patients previously underwent stenting using self-expanding stents. The study included 32 patients, with 25 tapered and seven non-tapered self-expanding stents deployed using a modified technique. Specifically, the stents were deployed from the V1 segment to the proximal subclavian artery [15].

The study analysis showed that the mean degree of stenosis decreased from 76.4% to 11.4% after stenting. No complications occurred during the procedure, and there were no vertebrobasilar strokes, transient ischemic attacks, or deaths over the 18.3 months of clinical follow-up. Only one patient experienced asymptomatic restenosis six months after the procedure during the mean angiographic follow-up of 12.5 months. A stent fracture did not occur, and there were no apparent events in the dependent upper extremity or issues with the involved subclavian artery. The researchers demonstrated the technical feasibility and safety of using self-expanding stents and contributed valuable insights into the use of endovascular techniques for the treatment of symptomatic VA-origin stenosis [15].

Dabus et al. conducted a study to assess the effectiveness of endovascular treatment in patients with symptomatic VBI stemming from the VA origin. The researchers analyzed clinical records, radiological reports, and procedural reports from 25 patients who underwent endovascular procedures. All patients presented with ischemic symptoms related to posterior circulation. They were unresponsive to antiplatelet drug therapy and had a digital subtraction angiogram indicating VA-origin stenosis of more than 50% [16].

The study results demonstrated that in 23 out of 25 patients, the contralateral VA was present with occlusion, hypoplasia, absence, or greater than 50% stenosis. The left VA was affected in 18 out of 25 patients, with a mean stenosis of 82.6%. A total of 28 procedures were performed, consisting of 23 angioplasty/stenting and five angioplasties alone. Overall, the endovascular treatment's success rate was 92.8%. There were no peri-procedural complications, including transient ischemic attack, stroke, or death. Follow-up records were available for 19 patients, with an average follow-up of 24 months. Five patients experienced recurrent symptoms of vertebrobasilar ischemia, and three required retreatment [16].

At the University Cardiovascular Clinic, Radak et al. demonstrated successful endovascular treatment of VA stenosis in 68 of 73 patients (93.2%). All procedures were conducted without the use of cerebral protection. During the follow-up period (mean of 44.3±31.2 months; range between two and 144 months), the primary patency rates at one, three, five, and seven years were 98.4%, 87.3%, 87.3%, and 87.3%, respectively. It should be noted that the researchers employed rigorous methodology in evaluating and treating all patients [17].

After diagnosing VA stenosis, each patient underwent evaluation and consultation with a neurologist and vascular surgeon. The best medical therapy was prescribed for all patients, except those with posterior circulation transient ischemic attack and patients with simultaneous carotid artery and VA near-total occlusion, who required immediate treatment. If symptoms did not improve under the best medical therapy for at least two months, an interdisciplinary group consisting of a vascular surgeon, neurologist, and interventional radiologist decided on endovascular treatment. These procedures were performed by interventional vascular specialists in an angiography suite [17].

Each consecutive patient received acetylsalicylic acid of 100 milligrams (mg) per day and either ticlopidine (250 mg twice daily) or clopidogrel (75 mg/d) three days before the intervention. After the intervention, all patients received dual therapy for 12 months, with acetylsalicylic acid (100 mg/d) continued. Statins were administered to 61 out of 73 patients (83.6%) upon discharge. Their approach was systematic and practical, aiming to avoid further complications in their patients [17].

A case series published by Mohammadian et al. further corroborated the efficacy of endovascular techniques in treating symptomatic VA stenosis patients. A total of 206 patients underwent the procedure, with an average baseline mRS score of 2.59±0.58 and a National Institutes of Health Stroke Scale (NIHSS) score of 7.94±2.6. A stent was successfully inserted in 199 (96.6%) patients. The technical success rate was 97.6%, leading to clinical success for all 206 symptomatic patients. Additionally, regardless of medical history, all patients received a daily prescription of atorvastatin, 20 mg. This treatment was administered for at least six months and continued for patients with hypercholesterolemia [18].

During the follow-up period, there were no reported deaths. The overall complication rate during follow-up was 6.3%, with transient ischemic attacks occurring in 4.4% of patients, a minor stroke in 1.4%, and a significant stroke in one patient. Impressively, the patient event-free survival rate reached 92.4%. These outcomes demonstrated the safety and feasibility of performing PTA, irrespective of a stent implant, leading to high technical success, minimal complications, low restenosis, and sustained clinical success for individuals with symptomatic VA stenosis [18].

Eberhardt et al. in a case series reported 20 patients with symptomatic high-grade vertebrobasilar stenoses who were unresponsive to drug therapy. Out of these 20 patients, nine had lesions in the V0 artery, two had lesions in the V3 artery, five had lesions in the V4 artery, and four had basilar artery lesions. The patients underwent vertebrobasilar stenting [19].

All cases achieved primary interventional success, with an average residual stenosis of 3% ± 4% in V0, 5% ± 4% in V3/4, and 7% ± 3% in basilar artery lesions. Patients with vertebral ostial lesions experienced no peri-interventional neurologic complications, transient ischemic attack, or stroke upon follow-up. However, patients with V4 or basilar artery lesions had two transient and three permanent clinical deteriorations, some of whom had acute stroke. The patency rate was 100% during the last examination. It is crucial to emphasize the significance of these findings, indicating that stenting the VBA is safe, with a low incidence of cerebral ischemic events during follow-ups. Although restenosis may occur, further comprehensive comparative trials are imperative [19].

In their non-randomized cohort study, Zi et al. aimed to determine the effectiveness of endovascular stents in patients with acute basilar artery occlusion (BAO). The study included only patients with confirmed radiological evidence of symptomatic and acute BAO. The researchers assessed 1254 patients and divided the eligible 829 patients into two groups: one group underwent endovascular stenting and received standard medical treatment, while the other group received standard therapy alone [20].

The results were highly encouraging, as endovascular stenting significantly improved the patients' functional outcomes at 90 days (p<0.001) compared to the standard treatment group. The intervention also showed a remarkable increase in the number of patients achieving mRS scores of 3 or less at 90 days (p<0.001) and a lower mortality rate (p<0.001) compared to the standard treatment group. Although there was an adverse effect, symptomatic intracerebral hemorrhage, it was relatively low, affecting only 7.1% of patients who underwent endovascular stenting alongside standard medical treatment, compared to 0.5% in the standard treatment group (p<0.001). Notwithstanding, overall, these findings indicate the potential benefits of incorporating endovascular stenting into managing acute BAO, offering patients new hope for improved outcomes [20].

In a prospective study spanning two years, Rangel-Castilla et al. enrolled 11 patients who presented with stenosis of VA ostium (VAO), with an average stenosis of 83.6%, confirmed on neuroimaging/Doppler studies. Only nine patients exhibited contralateral VAO stenosis between 40% and 60%. Their pre-surgical average mRS score was 1.25. All study patients, having previously failed to respond to medical therapy, underwent stenting and dual balloon Flash angioplasty [21].

Following the surgery, the researchers reported stent apposition against the VA and around the ostium in the subclavian artery without damage. There were no reported permanent peri-operative complications or deaths. After an average follow-up of 10.8 months (ranging from 2 to 24), all patients experienced symptom resolution, and on neuroimaging or Doppler studies, there were no signs of symptomatic restenosis. The average mRS score was 0.66, indicating a positive outcome [21].

Jin et al. conducted a study on evaluating and treating patients with intradural vertebrobasilar dissecting aneurysms using endovascular techniques. The study included a total of 42 patients, out of which 29 had ruptured aneurysms and 13 had unruptured dissecting aneurysms. The endovascular modalities used in the study included trapping, proximal occlusion, stent with coil, and stent alone. Specifically, 30 patients underwent trapping, three underwent proximal occlusion, six had a stent with the coil, and three had a stent alone [22].

Endovascular treatment was successful for 17 out of the 29 patients with ruptured vertebrobasilar dissecting aneurysms, with no procedural complications. However, some complications occurred during the medical procedures. These complications included three cases of rebleeding, six cases of posterior inferior cerebellar artery territory infarction, two cases of brain stem infarction, and one case of thromboembolism-related multiple infarctions. Despite these complications, out of 42 patients, 32 experienced positive results, with the researchers reporting that 76.2% of patients treated for vertebrobasilar dissecting aneurysms had favorable clinical outcomes. However, there were some deaths associated with the procedures. Three procedure-related deaths were due to rebleeding, and one non-procedure-related death was due to pneumonia sepsis [22].

Notably, all 13 patients with unruptured vertebrobasilar dissecting aneurysms experienced favorable clinical and radiologic outcomes and did not experience any procedure-related complications. This shows that endovascular treatment can be viable for patients with this condition. Still, it is crucial to consider the potential risks and complications during medical procedures [22].

In a prospective, single-center trial, Fan et al. compared the safety and effectiveness of endovascular techniques for treating acute ischemic stroke caused by the blockage of a large artery in the posterior circulation. The study included 67 patients with acute vertebrobasilar occlusion, with a confirmed diagnosis by either CTA or MRA. All patients had a baseline NIHSS score ≥2, premorbid mRS score ≤2, and posterior circulation acute stroke prognosis early computed tomography score ≥6 [23].

Of the 67 patients, 35 (52.2%) had underlying intracranial atherosclerosis (ICAS), while 32 (47.8%) did not. The researchers found rescue therapies were more commonly performed in the ICAS group (82.9% versus 34.4%; p=0.000). Moreover, patients in the ICAS group had more favorable outcomes at 90 days than those in the non-ICAS group (71.4% versus 46.9%; p=0.041). Both groups showed no significant variation concerning symptomatic intracranial hemorrhage or death within 90 days. However, the study demonstrated that the baseline Glasgow Coma Scale scores (p=0.004) and pons-midbrain index scores (p=0.001) were independently associated with favorable outcomes at 90 days [23].

The results of the study suggest that endovascular therapy with stent-retriever thrombectomy followed by rescue treatment can effectively manage acute ischemic stroke caused by posterior circulation large artery occlusion. This approach can help achieve high rates of successful revascularization and favorable outcomes [23].

A randomized controlled trial conducted by Langezaal et al. evaluated the effectiveness of endovascular therapy in treating patients who had a stroke caused by BAO. The study included 300 patients who had a stroke due to BAO, and the patients were randomly assigned to two groups, the endovascular therapy group and the standard medical care group, in a 1:1 ratio [24].

The study's primary outcome was a favorable functional outcome based on mRS scores at 90 days. Researchers also assessed the primary safety outcomes, which were symptomatic intracranial hemorrhage within three days after the initiation of treatment and mortality at 90 days. The researchers found that 78.6% of the patients in the endovascular group received intravenous thrombolysis, while 79.5% of those in the medical care group received the same. Endovascular treatment was initiated at a median of 4.4 hours after stroke onset [24].

The study revealed that 44.2% of patients in the endovascular group and 37.7% of those in the medical care group had a favorable functional outcome (risk ratio, 1.18; 95% confidence interval (CI), 0.92-1.50). Although there was no significant difference between the two groups, the trial results did not exclude a substantial benefit of endovascular therapy. Nonetheless, symptomatic intracranial hemorrhage occurred in 4.5% of the patients after endovascular treatment compared to 0.7% in the medical care group (risk ratio, 6.9; 95% CI, 0.9-53.0), indicating that more extensive trials are needed to determine the efficacy and safety of endovascular therapy for BAO [24].

Olthuis et al. conducted a clinical trial, formally known as "MR CLEAN-LATE," to evaluate the safety and efficacy of endovascular treatment for patients presenting between six and 24 hours from symptom onset or when the patient was last seen well. The researchers based their selection criteria on the presence of collateral flow on CTA [25].

The MR CLEAN-LATE trial was a multicenter, open-label, blinded-endpoint, randomized, controlled study that involved 18 stroke intervention centers. The researchers included patients aged 18 years or older with ischemic stroke who presented in the late window with an anterior circulation large-vessel occlusion, collateral flow on CTA, and a neurological deficit score of at least 2 on NIHSS. Patients eligible for late-window endovascular treatment (24 hours beyond the initial symptoms presented) were treated according to national guidelines and excluded from the MR CLEAN-LATE enrolment. The researchers randomly assigned patients in a 1:1 ratio to receive either endovascular or no endovascular treatment (control) and the best medical treatment [25].

The primary outcome was the mRS score after 90 days of randomization. The researchers also included safety outcomes such as all-cause mortality 90 days after randomization and symptomatic intracranial hemorrhage. All randomly assigned patients who provided deferred consent or died before consent could be obtained comprised the modified intention-to-treat population, in which the primary and safety outcomes were assessed. The researchers adjusted their analyses for predefined confounders. The treatment effect was estimated with ordinal logistic regression and reported as an adjusted standard OR with a 95% CI [25].

Five hundred and thirty-five patients were enrolled in a study, of which 502 (94%) either provided deferred consent or passed away before consent could be obtained. The patients were divided into two groups, the endovascular treatment group (255 patients) and the control group (247 patients), with females constituting 52% of the total population. The median mRS score at 90 days was found to be lower in the endovascular treatment group when compared to the control group (3 (interquartile range 2-5) versus 4 (2-6)). The endovascular treatment group showed a shift towards better outcomes on the mRS, as observed by the researchers, with an adjusted joint OR of 1·67 (95% CI 1.20-2.32) [25].

There was no significant difference in all-cause mortality between the two groups. However, the frequency of symptomatic intracranial hemorrhage was higher in the endovascular treatment group compared to the control group. Specifically, 17 patients (7%) experienced symptomatic intracranial hemorrhage in the endovascular group, while only four (2%) experienced it in the control group. The adjusted odds ratio was 4.59 (95% CI 1.49-14.10). Nevertheless, the MR CLEAN-LATE trial demonstrated that endovascular treatment was effective and safe in treating patients with ischemic stroke caused by anterior circulation large-vessel occlusion who presented between six and 24 hours from the onset of symptoms [25].

In a multinational, multicenter, randomized controlled trial, open-label with a blinded endpoint, Mitchell et al. not only established the effectiveness of the direct endovascular procedure in treating large-vessel occlusion but also showed that its outcomes are comparable to endovascular procedures with a standard bridging therapy. Bridging therapy refers to administering thrombolytics before performing the endovascular thrombectomy [26].

The study team recruited adult participants who suffered from a stroke with a blockage in the intracranial internal carotid artery, middle cerebral artery, or basilar artery. The occlusion was confirmed by non-contrast CT and vascular imaging. The patients were required to present within 4.5 hours of experiencing a stroke. The researchers randomly allocated eligible participants in a 1:1 ratio to undergo direct endovascular thrombectomy (direct group) or bridging therapy followed by endovascular thrombectomy. Patients in the bridging therapy group received intravenous thrombolytic treatment (alteplase or tenecteplase) as per standard care before undergoing endovascular thrombectomy. To evaluate the efficacy of the treatment, the researchers defined the primary endpoint as functional independence at 90 days, measured by a mRS score of 0-2 or return to baseline. They analyzed the intention-to-treat risk difference and set a non-inferiority margin of -0.1 [26].

Of 295 patients randomly assigned to the two study groups, 148 underwent direct endovascular thrombectomy, and 147 received bridging therapy followed by endovascular thrombectomy. The results indicated that 55% of the 146 patients in the direct group achieved functional independence (the data on two patients in the direct group was not reported due to undisclosed reasons), while 61% of the 147 patients in the bridging group achieved the same outcome. The intention-to-treat risk difference was -0.051, with a two-sided 95% CI of -0.160 to 0.059. The per-protocol risk difference was -0.062, with a two-sided 95% CI of -0.173 to 0.049 [26].

The direct group and the bridging group had similar safety outcomes. The direct group had two cases of symptomatic intracerebral hemorrhage out of 146 patients, while the bridging group had only one case out of 147 patients. Similarly, there was no remarkable difference in the number of patients who died in both groups. Out of 146 patients in the direct group, 22 (15%) passed away. In the bridging group, 24 patients (16%) died out of 147. The adjusted odds ratio for symptomatic intracerebral hemorrhage was 1.70 (95% CI 0.22-13.04), and for death, it was 0.92 (95% CI 0.46-1.84). The study showcased that patient outcomes of direct endovascular thrombectomy were comparable to those of endovascular thrombectomy with standard bridging therapy [26].

Please refer to Table 2 for the key findings of the reviewed studies highlighting endovascular repair in treating patients with VIB.

**Table 2:** Key studies highlighting the effectiveness of endovascular treatments in treating patients with vertebrobasilar insufficiency. The table summarizes the findings of the current literature on the effectiveness of endovascular intervention in treating patients with vertebrobasilar insufficiencies. All the studies mentioned in the table met the inclusion criteria of this systematic review. %: percentage; VA: vertebral artery; VBA: vertebrobasilar artery

Authors	Type of study	Sample size	Findings
Antoniou et al. (2012) [12]	Retrospective study	1099	High success rate (97%) and low adverse effects of endovascular treatment in patients with proximal VA stenosis. Dual antiplatelet therapy was used before and after the procedure.
Liu et al. (2020) [13]	Randomized controlled trial	288	Endovascular surgical approach was effective and safe for treating VBA occlusion. Comparable outcomes to standard treatment, with no statistical difference in functional independence rates.
Kiyosue et al. (2004) [14]	Case report	2	Successful treatment of restenosis after endovascular stenting, with potential to enhance patient outcomes and offer a safer surgical approach.
Li et al. (2014) [15]	Retrospective study	32	Technical feasibility and safety of self-expanding stents for VA-origin stenosis treatment. Significant decrease (from 76.4% to 11.4%) in the mean degree of stenosis and no vertebrobasilar strokes or complications reported.
Dabus et al. (2006) [16]	Observational study	25	High success rate (92.8%) of endovascular treatment for symptomatic vertebral basilar insufficiency originating from the VA. No peri-procedural complications such as transient ischemic attack, stroke, or death reported.
Radak et al. (2014) [17]	Prospective study	73	Consistently high primary patency rates (93.2%) following endovascular treatment for VA stenosis. Patients received combination medical therapies before the intervention, as determined by an interdisciplinary team.
Mohammadian et al. (2013) [18]	Case series	206	High technical success rate (97.6%) and safety of percutaneous transluminal angioplasty with or without stenting for symptomatic VA stenosis. No reported deaths during follow-up, indicating a patient event-free survival rate of 92.4%.
Eberhardt et al. (2006) [19]	Case series	20	Safety and efficacy of endovascular treatment in patients with symptomatic high-grade vertebrobasilar stenoses. No peri-interventional complications or strokes, but further comparative trials needed to ensure safety and prevent restenosis.
Zi et al. (2020) [20]	Non-randomized controlled trial	1254	Potential benefits of endovascular stenting in managing acute basilar artery occlusion, with improved outcomes and hope for recovery. Higher incidence of symptomatic intracerebral hemorrhage in the stenting group compared to the standard treatment group.
Rangel-Castilla et al. (2016) [21]	Prospective study	11	Symptom resolution and no symptomatic restenosis observed following stenting and dual balloon Flash angioplasty in patients with VA-origin stenosis. Improvement in the average modified Rankin Scale score suggested a positive outcome.
Jin et al. (2009) [22]	Prospective study	42	Endovascular treatment for vertebrobasilar dissecting aneurysms resulted in favorable outcomes for most patients (76.2%). Seventeen out of 29 patients with ruptured aneurysms were successfully treated with no complications. All 13 patients with unruptured aneurysms also had favorable outcomes without any complications. However, there were some complications and a few deaths.
Fan et al. (2019) [23]	Prospective study	67	The study found that patients with acute ischemic stroke due to underlying intracranial atherosclerosis had more favorable outcomes (71.4%) with endovascular procedures. The results suggested that endovascular therapy can effectively manage acute ischemic stroke caused by posterior circulation large artery occlusion.
Langezaal et al. (2021) [24]	Randomized controlled trial	300	Endovascular surgical options did not significantly differ from standard medical care regarding favorable functional outcomes for patients. However, there was a possibility of a substantial benefit of endovascular therapy. Symptomatic intracranial hemorrhage occurred more frequently in the endovascular treatment group (4.5%) than in the medical care group (0.7%).
Olthuis et al. (2023) [25]	Randomized controlled trial	502	The endovascular treatment group had a lower median modified Rankin Scale score at 90 days and showed a shift towards better outcomes. However, there was no significant difference in all-cause mortality between the two groups. The endovascular treatment group had a higher frequency of symptomatic intracranial hemorrhage compared to the control group.
Mitchell et al. (2022) [26]	Randomized controlled trial	295	The patient outcomes of direct endovascular thrombectomy were comparable to those of endovascular thrombectomy with standard bridging therapy.

Discussion

VBI is a significant medical condition caused by the lack of adequate blood flow to the posterior circulation of the brain. It is often associated with VBA stenosis or occlusion, leading to devastating consequences such as stroke, cognitive impairment, and even death [2]. An endovascular approach has been explored as a treatment option for patients with VBI caused by stenosis or occlusion. This paper reviewed relevant research studies conducted in the last 20 years, focusing on the efficacy, safety, and potential implications of endovascular interventions for treating VBI.

The endovascular approach in treating VBI shows promising results in terms of efficacy and safety, with some methodological limitations and inconsistencies that warrant further research. Several studies have reported high success rates and low adverse events following endovascular treatment for VBI. For example, Antoniou et al. reported a 97% success rate with a low rate of complications [12], while Li et al. demonstrated the technical feasibility and safety of using self-expanding stents for VA-origin stenosis treatment [15]. Similarly, Mohammadian et al. and Radak et al. also reported high technical success rates and low complication rates after endovascular therapy [17,18].

In most studies, patients were pretreated with dual antiplatelet therapy and followed up for a varying duration. During the follow-up period, some studies reported the occurrence of restenosis and the need for reintervention. For example, Antoniou et al. reported a 23% restenosis rate in patients with follow-up imaging [12], while Li et al. found an asymptomatic restenosis rate of 3.1%. The research findings suggest that endovascular intervention can be an effective and safe treatment option for VBI. There is the potential to improve patient outcomes through the development of new techniques, such as the use of self-expanding stents [15] and endovascular stenting in acute BAO [20]. Stenting in VBA has demonstrated excellent patency rates without significant complications, hinting that these procedures could offer better outcomes for patients with the disease and reduce stroke or other severe consequences of VBI [19].

There are several limitations and inconsistencies in the reviewed studies. Most investigations have relatively small sample sizes, which can impact the statistical power and generalizability of the findings. Another limitation is the variability in the study design, as some studies are retrospective [12,15]. In contrast, others are prospective [17,21-23] or randomized controlled clinical trials [13,24-26].

The follow-up period durations also vary, which can affect the clinical applicability of the results. Restenosis rates and complication rates differ between studies, which may result from differences in patient populations, study designs, and endovascular techniques used. Additionally, most studies do not report long-term outcomes, making it challenging to evaluate the durability and sustainability of endovascular interventions for VBI [12-17]. Expanding research in this area is essential to address these limitations and inconsistencies. Future studies should aim to have larger sample sizes, standardized study designs, more extended follow-up periods, and comprehensive outcomes reporting. Furthermore, there is a gap in research on endovascular interventions for VBI in specific patient populations, such as elderly individuals or those with comorbidities.

Moreover, while endovascular stenting has shown promising results in acute BAO, there is currently limited research on its use in chronic VBI cases [20]. This highlights the need for further investigation and direct comparison of endovascular interventions with other treatment modalities, such as medical management or surgical procedures, including open endarterectomy. Moreover, incorporating patient perspectives and quality-of-life measures in research will also provide valuable insight into the patient experience and satisfaction with endovascular interventions for VBI. This can help guide treatment decisions and improve overall patient care.

## Conclusions

Endovascular treatments have shown significant technical success rates in treating VBI. Mortality rates are considerably low, along with minor adverse effects such as intracranial hemorrhage and restenosis. The overall incidence rate of these complications is low. Combination medical therapies, when used along with endovascular intervention, have demonstrated improved outcomes and reduced restenosis rates. However, methodological limitations and inconsistencies in the current literature require further investigation. Critical areas for future research include larger, randomized clinical trials and direct comparisons to other treatment options. Additionally, incorporating patient perspectives and quality-of-life measures in future studies can further enhance understanding and improve patient care in VBI treatment.
